# SARS-CoV-2 Cellular Entry Is Independent of the ACE2 Cytoplasmic Domain Signaling

**DOI:** 10.3390/cells10071814

**Published:** 2021-07-17

**Authors:** Thankamani Karthika, Jeswin Joseph, V. R. Akshay Das, Niranjana Nair, Packirisamy Charulekha, Melvin Daniel Roji, V. Stalin Raj

**Affiliations:** Virology Scientific Research (VSR) Laboratory, School of Biology, Indian Institute of Science Education and Research Thiruvananthapuram, Kerala 695551, India; karthika17@iisertvm.ac.in (T.K.); jeswinjoseph18@iisertvm.ac.in (J.J.); akshaydas19@iisertvm.ac.in (V.R.A.D.); niranjananair199917@iisertvm.ac.in (N.N.); charumarch1517@iisertvm.ac.in (P.C.); melvin17@iisertvm.ac.in (M.D.R.)

**Keywords:** SARS-CoV-2, SARS-CoV-1, coronavirus entry, ACE2, ACE2 internalization

## Abstract

Recently emerged severe acute respiratory syndrome coronavirus (SARS-CoV)-1 and -2 initiate virus infection by binding of their spike glycoprotein with the cell-surface receptor angiotensin-converting enzyme 2 (ACE2) and enter into the host cells mainly via the clathrin-mediated endocytosis pathway. However, the internalization process post attachment with the receptor is not clear for both SARS-CoV-1 and -2. Understanding the cellular factor/s or pathways used by these CoVs for internalization might provide insights into viral pathogenesis, transmission, and development of novel therapeutics. Here, we demonstrated that the cytoplasmic tail of ACE2 is not essential for the entry of SARS-CoV-1 and -2 by using bioinformatics, mutational, confocal imaging, and pseudotyped SARS-CoVs infection studies. ACE2 cytoplasmic domain (cytACE2) contains a conserved internalization motif and eight putative phosphorylation sites. Complete cytoplasmic domain deleted ACE2 (∆cytACE2) was properly synthesized and presented on the surface of HEK293T and BHK21 cells like wtACE2. The SARS-CoVs S1 or RBD of spike protein binds and colocalizes with the receptors followed by internalization into the host cells. Moreover, pseudotyped SARS-CoVs entered into wtACE2- and ∆cytACE2-transfected cells but not into dipeptidyl peptidase 4 (DPP4)-expressing cells. Their entry was significantly inhibited by treatment with dynasore, a dynamin inhibitor, and NH_4_Cl, an endosomal acidification inhibitor. Furthermore, SARS-CoV antibodies and the soluble form of ACE2-treated pseudotyped SARS-CoVs were unable to enter the wtACE2 and ∆cytACE2-expressing cells. Altogether, our data show that ACE2 cytoplasmic domain signaling is not essential for the entry of SARS-CoV-1 and -2 and that SARS-CoVs entry might be mediated via known/unknown host factor/s.

## 1. Introduction

The current COVID-19 pandemic, caused by the novel severe acute respiratory syndrome coronavirus 2 (SARS-CoV-2), has gained enormous attention for the development of novel therapeutics due to the high transmissibility and severity of disease manifestations [1,2]. Prior to the SARS-CoV-2 outbreak in 2019, two major human coronaviruses (CoVs), SARS-CoV-1 and the Middle East respiratory syndrome (MERS) CoV have affected the public in 2003 and 2012 respectively [3,4]. However, SARS-CoV-2 exhibits rapid transmission, severe respiratory illness, and high mortality rates in humans compared to the previously reported human CoVs [5]. As of 14th June 2021, 175 million laboratory confirmed cases and 3.7 million deaths have been reported globally, and it is continuing to cause outbreaks [6]. The increasing number of cases raises the importance for proper understanding of the virus–host interactions and is essential to develop novel therapeutics against COVID-19.

Similar to SARS-CoV-1 and MERS-CoV, SARS-CoV-2 belongs to the *Coronaviridae* family, with a positive-sense RNA genome of approximately 30 kb in size, and infects a wide range of birds and mammals, including humans [7]. The primary determinant of coronavirus infection and tropism is the virus spike glycoprotein, which binds to the host cell surface receptor/s and mediates the entry [8]. The spike protein of SARS-CoVs, a type I transmembrane fusion protein, is trimeric and highly glycosylated (Figure S1) [9]. The spike composed of approximately 1250 amino acids contains two subunits, S1 and S2 (Figure S1), and is localized on the surface of the coronavirus particle [9]. The N-terminal region contains the receptor-binding domain (RBD) and the C-terminal region has the fusion domain. SARS-CoV-2 initiates the infection by binding of the S1 subunit with its cellular receptor followed by the viral membrane fusion to the host cell, which is mediated through its S2 domain consisting of a heptad repeat (HR) region (Figure S1) [9]. Receptor interaction and subsequent endocytosis enable the virus to release its genome to the cytoplasm and thereby promote viral replication and assembly.

**Figure 1:**
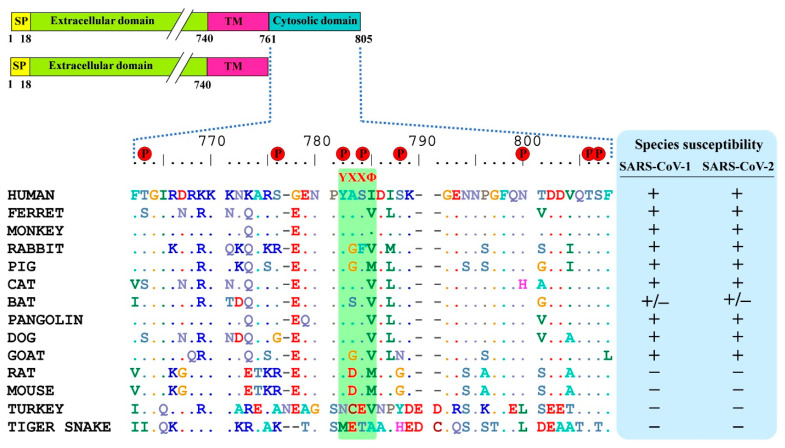
Comparison of conservative residues in ACE2 cytoplasmic domain of different species. Sequences were retrieved from NCBI and multiple sequence alignment was performed using the BioEdit software. Predicted putative phosphorylation sites are indicated by red circle. Susceptible and non-susceptible species to SARS-CoV-1 and -2 are denoted in ‘+’ and ‘−’signs respectively.

SARS-CoVs interact with the host cell receptor angiotensin-converting enzyme 2 (ACE2) and enter the cells, majorly employing the host cell endocytic pathways [10,11]. ACE2, a type I transmembrane protein which regulates the blood pressure, is majorly distributed in the epithelial cells of the lung, heart, intestine, kidney, and alveolar tissues [12]. ACE2 from different species, such as amphibians, birds, and mammals, share a conserved primary structure [13]. Binding of SARS-CoV-2 spike on ACE2 triggers cleavage of the spike at the S1–S2 proteolytic site by proteases such as TMPRSS2/4, furin, and/or Cathepsin B/L, and mediates the fusion of the viral membrane either at the plasma membrane or at the endosomal compartment [14]. In addition, recent evidence shows that the entry of SARS-CoV-2 is promoted by additional host factors such as AXL, Neuropilin-1 (NRP-1), Basigin (CD147), AT1(Angiotensin II receptor type 1), and AVPR1B (Vasopressin V1b receptor) proteins [15,16,17,18]. However, the dynamics of ACE2-mediated signaling mechanisms for recruiting multiple host factors to aid the SARS-CoV-2 internalization are not well understood. In general, for cell-surface receptors, once the ligand binds to the extracellular domain, a signal is transmitted to the intracellular domain and initiates the entry process [19]. Like other membrane receptors, ACE2 contains a long extracellular domain (740 aa), a 20 aa transmembrane, and a short 43 aa cytoplasmic domain [11] (Figure 1), but the mechanism of signal transmission through the cytoplasmic domain of ACE2 upon virus binding has not been explored yet. Understanding the role of the cytoplasmic domain of ACE2 will elucidate the role of other adapter proteins involved in SARS-CoV-2 entry and promote the identification of novel therapeutic targets against SARS-CoV-2. In this study, we evaluate the role of the cytoplasmic domain of ACE2 in the entry of SARS-CoV-2.

## 2. Materials and Methods

### 2.1. Cell Lines

Human embryonic kidney 293T cells (HEK293T-ATCC CRL-3216, Manassas, VA, USA) and African green monkey kidney cells (Vero E6-ATCC CRL-1586, Manassas, VA, USA) were maintained in Dulbecco’s modified Eagle medium (Lonza, Cat No.12604F, Walkersville, MD, USA). Baby hamster kidney cells (BHK21-ATCC CCL-10, Manassas, VA, USA) were grown in Eagle’s minimal essential medium (Lonza, Cat No.12-611F, Walkersville, MD, USA) and the human liver cell line (Huh7 cells were obtained from Erasmus Medical Centre, Rotterdam, The Netherlands) was maintained in RPMI-1640 media (Lonza, Cat No-12115F, Walkersville, MD, USA). All cell lines were supplemented with 10% fetal bovine serum (MP Biomedicals, Cat No.29101, Solon, OH, USA) and 1% penicillin/streptomycin (Lonza, Walkersville, MD, USA) and were grown at 37 °C in a CO_2_ incubator.

### 2.2. Bioinformatic Analysis of ACE2 Sequences

Amino acid sequences of ACE2 of different species were retrieved from the UniProt database (accession numbers: Q9BYF1, Q2WG88, A0A0D9RQZ0, G1TEF4, K7GLM4, Q5EGZ1, Q8R0I0, Q56H28, and U5WHY8) and subjected to multiple sequence alignment using BioEdit software, and the sequence conservation of the cytoplasmic domain of ACE2 between distinct species was analyzed. The internalization motifs within the cytoplasmic tail were manually identified as described elsewhere [20]. The putative phosphorylation sites of the ACE2 cytoplasmic domain were predicted through NetPhos 3.1. software.

### 2.3. Generation of Plasmid Constructs

The full-length ACE2 (805 aa) gene was amplified from cDNA of Huh-7 cells and cloned into a pcDNA3.1(+) eukaryotic expression plasmid (pcDNA-ACE2). To produce soluble ACE2 protein, ACE2 gene was polymerase chain reaction (PCR) amplified from pcDNA-ACE2 without a transmembrane domain and inserted between KpnI and XhoI sites in a pCAGGS expression vector (pCAGGS-sACE2). Similarly, the conventional PCR method was used to generate the ACE2 cytoplasmic domain deletion mutant (Δ 762-805) in pcDNA3.1 vector (pcDNA3.1-∆cytACE2).

Next, the full-length spike of MERS-CoV (GenBank: JX869059, 1321 aa), SARS-CoV-1 (GenBank: AY278491, 1236 aa), and SARS-CoV-2 (GenBank: MN908947, 1254 aa) lacking the endoplasmic retention signal were amplified either from cDNA or from synthetic constructs and sub cloned into pCAGGS expression plasmid (pCAGGS-MERS-S, pCAGGS-SARS-1-S, and pCAGGS-SARS-2-S) [21]. Finally, for the generation of Fc fusion constructs, spike S1 domains of MERS-CoV (747 aa), SARS-CoV-1 (676 aa), and SARS-CoV-2 (683 aa) were C-terminally fused with the Fc domain of human immunoglobulin G (IgG) and cloned into a pCAGGS vector (pCAGGS-MERS-S1-Fc, pCAGGS-SARS-1-S1-Fc, and pCAGGS-SARS-2-S1-Fc). Similarly, RBD of MERS-CoV (residues 358-588), SARS-CoV-1 (residues 318-527), and SARS-CoV-2 (residues 340-550) was PCR amplified from the spike S1 gene of respective plasmids and subcloned into pCAGGS expression plasmid (pCAGGS-MERS-RBD-Fc. pCAGGS-SARS-1-RBD-Fc, and pCAGGS-SARS-2-RBD-Fc).

### 2.4. Production of Recombinant Proteins

Spike S1-Fc or RBD-Fc proteins of SARS-1, SARS-2, and MERS- CoVs, and soluble ACE2 were produced by transiently transfecting HEK293T cells with recombinant plasmid constructs of pCAGGS-MERS-S1-Fc, pCAGGS-SARS-1-S1-Fc, pCAGGS-SARS-2-S1-Fc, pCAGGS-MERS-RBD-Fc, pCAGGS-SARS-1-RBD-Fc, pCAGGS-SARS-2-RBD-Fc, or pCAGGS-sACE2. Five days post transfection, supernatants were harvested and the cell debris was removed by centrifugation at 1200 rpm for 10 min. The recombinant proteins were purified using Protein A sepharose affinity chromatography (GE Healthcare) following the protocol described elsewhere [22]. Similarly, soluble ACE2 was purified using Nickel-NTA agarose beads (Qiagen, Cat No. 1018244, Hilden, Germany) and eluted with 200 mM imidazole. The quality and quantity of the protein were measured by Nanodrop (Denovix, Wilmington, DE, USA), SDS-PAGE, and Western blot analysis.

### 2.5. Analysis of ACE2 Transcript by Reverse Transcription Polymerase Chain Reaction (RT-PCR)

**Figure 2:**
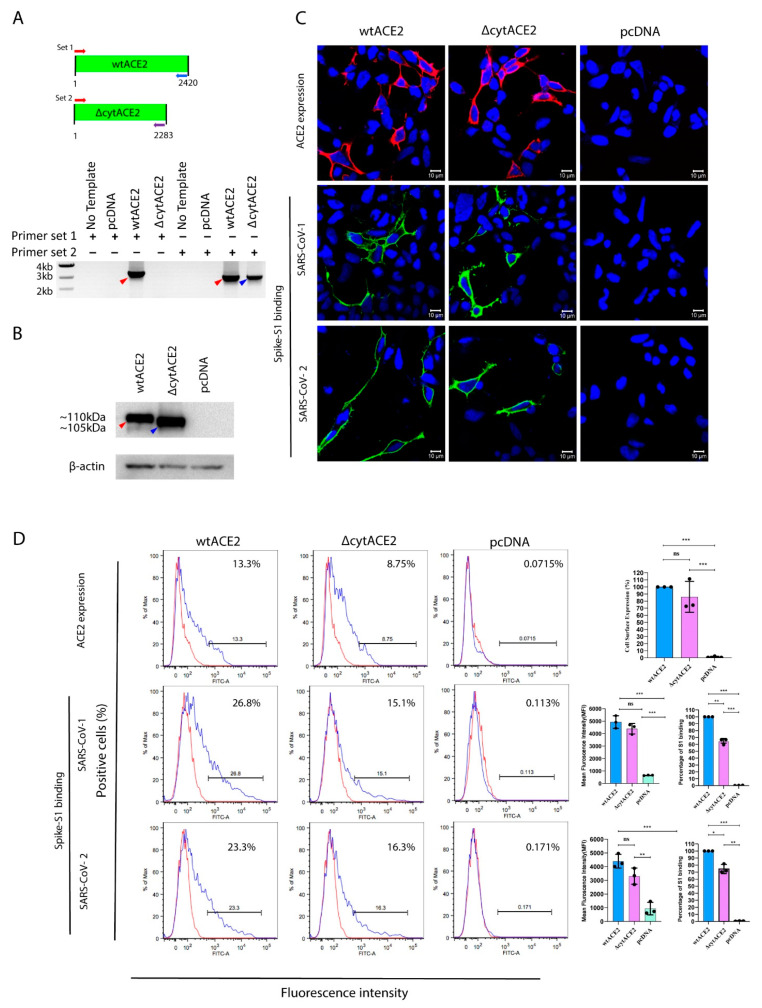
Cell surface expression and binding of SARS-CoV spike S1 proteins on wtACE2 or ΔcytACE2 receptor. Confirmation of expression of wtACE2 and ∆cytACE2 in HEK293T-transfected cells. (**A**) Total RNA was extracted from the cells transfected with either wtACE2 or ΔcytACE2 and RNA transcripts were analyzed using RT-PCR by specific primer sets. (**B**) Protein expression was confirmed by Western blot analysis of cell lysates. Red arrow indicates wtACE2 and blue indicates ΔcytACE2 (**C**) Confocal imaging and quantitative flowcytometry analysis of wtACE2 and ΔcytACE2 surface expression on HEK293T cells and SARS-CoVs S1-Fc binding. Plasmids encoding wtACE2 and ∆cytACE2 were transiently expressed in HEK293T cells and the ACE2 expressions were detected using anti-ACE2 polyclonal antibody at 24 h post transfection (red, upper panel). Similarly, cells expressing wtACE2 and ∆cytACE2 were treated with either SARS-CoV-1 or -2 S1-Fc proteins (5 µg/mL) and stained using goat anti human IgG Fc conjugated with FITC (green, middle, and lower panels). Nuclei were stained with DAPI (blue). (**D**) Cell surface expression of wtACE2 and ∆cytACE2 on HEK293T cells (upper panel), and S1 binding on wtACE2 and ∆cytACE2 receptors (middle and lower panels). Quantitative and comparative analysis of wtACE2 and ΔcytACE2 expression and S1-Fc binding on HEK293T cells (right graph). Data represented as mean fluorescence intensity and the percentage of S1-binding cells. Data are shown as mean ± SD. *p* value was determined by one-way ANOVA analysis (* *p* < 0.05; ** *p* < 0.01; *** *p* < 0.001).

An RT-PCR assay was performed to validate the complete deletion of the cytoplasmic domain of ACE2 receptor in BHK21 cells. Briefly, confluent BHK21 cells were transfected with 3 μg of plasmids encoding either wildtype (wt) ACE2 or ∆cytACE2. At 24 h post transfection, cells were harvested and total RNA was extracted using a Qiagen tissue RNA isolation kit (Cat No.74104, Hilden, Germany) according to the manufacturer’s instructions. Complementary DNA (cDNA) was synthesized using SuperScript IV reverse transcriptase (ThermoFisher Scientific, Cat No.18090050, Vilnius, Lithuania) and complete and 43 aa cytoplasmic tail-deleted ACE2 genes were PCR amplified using combinations of specific primer sets (set1: Fwd—5′-TATGGTACCACCATGTCAAGCTCTTCCTGGCT-3′, Rev—5′-TATGGTACCCTATAAAAGGAGGTCTGA-3′ and set2: Fwd—5′TATGGTACCACCATGTCAAGCTCTTCCTGGCT-3′, Rev—5′-TATTCTAGACTAGATCAGGATGACAATGCCAA-3′), as shown in Figure 2A.

### 2.6. SDS-PAGE and Western Blot Analysis

Expression of spike S1-Fc and RBD-Fc of SARS-1, SARS-2, and MERS- CoVs were analyzed by SDS-PAGE and Western blot as described elsewhere [23]. Briefly, purified recombinant proteins were subjected to 10 min boiling and resolved in 10% Tris-glycine SDS-PAGE and stained with Coomassie brilliant blue R-250 or transferred to nitrocellulose membranes. Next, the membrane blots were blocked with 5% skim milk in 1x Tris-buffered saline tween (TBST) for 1 h at room temperature (RT) and incubated further with goat anti-human IgG Fc antibodies conjugated with horseradish peroxidase (HRP) (Bethyl, 1:5000, Cat No. A80-119P, Montgomery, TX, USA) for 1 h at RT, and then the bands were visualized with ECL Western blot substrate reagents (Bio-Rad, Cat No.170-5060, Hercules, CA, USA). Similarly, expression of sACE2 was confirmed with Coomassie staining and Western blot using goat anti-human ACE2 polyclonal antibody (R&D, Cat No. AF933, Minneapolis, MN, USA).

### 2.7. Immunofluorescence Analysis of wtACE2 and ∆cytACE2 Cell Surface Expression

BHK21 cells were grown on coverslips at 70% confluency and transfected with 2 µg expression plasmids of either pcDNA3.1-ACE2, pcDNA3.1-∆cytACE2, or pcDNA3.1 independently. At 4 h post-transfection, cells were replaced with 1% FBS containing EMEM and maintained at 37 °C for 24 h [23]. Coverslips were washed with 1× PBS and fixed with 4% paraformaldehyde. Then, cells were washed twice with PBS and blocked with 1% bovine serum albumin (BSA) for 1 h. Immunofluorescence staining was performed using goat anti-ACE2 antibody (R&D, Cat No.AF933, 1:300, Minneapolis, MN, USA) for 1 h. Next, the cells were subjected to PBS wash twice and stained with rabbit anti-goat antibody conjugated with Alexa Fluor 594 (Immunotag, Cat No. ITIF59418, 1:500, St. Louis, MO, USA) followed by 4′,6-diamidino-2-phenylindole (DAPI) staining. Coverslips were fixed and imaging analysis was performed using a Zeiss LSM 880 confocal laser scanning microscope (Zeiss, Jena, Germany).

### 2.8. Quantification of wtACE2 and ∆cytACE2 Expression by Flow Cytometry

Surface expression of wtACE2 and ∆cytACE2 were analyzed by flow cytometry as described elsewhere [23]. Briefly, 2 µg of recombinant pcDNA-ACE2, pcDNA-∆cytACE2, or pcDNA were transfected in BHK21 cells seeded in a 24 well plate at 1 × 10^5^ cells per well. At 24 h post transfection, cells were washed with 1× PBS, harvested after trypsin treatment, and resuspended in ice-cold PBS. Further, anti-ACE2 antibody was added to the cells (5 μg/mL) and incubated for 1 h at 4 °C. Following incubation, cells were washed twice with ice-cold PBS and stained using secondary antibody conjugated with Alexa Fluor 594 for 1 h at 4 °C. After incubation, cells were washed twice and resuspended in 200 μL of PBS and analyzed using a flow cytometer (FACS BD Aria III, San Jose, CA, USA). ACE2 expression was quantified by mean fluorescence intensity (MFI) data using Flowjo software v.10. MFI values of negative control were subtracted from all samples and expression of wtACE2 was set as 100% for normalization. Relative surface expression of ∆cytACE2 was calculated with respect to wtACE2 expression.

### 2.9. Coronavirus Spike Protein-Binding Assay on ∆cytACE2

To evaluate the binding interaction of wildtype and mutant receptors with spike S1-Fc proteins, we performed a binding assay in BHK21 cells [23]. Wildtype and mutant plasmids were transfected in BHK21 cells (1 × 10^5^ density per well) in a 24-well plate. At 24 h post transfection, cells were harvested after trypsin treatment, and washed with 1× PBS. Cells were incubated with spike S1-Fc proteins of SARS-CoV-1 and -2 (5 µg/mL) for 1 h at 4 °C. After incubation, cells were washed twice with ice-cold PBS, and S1-Fc was stained with goat anti-human IgG-Fc antibody conjugated with FITC (Bethyl, Cat No. A80119F, 1:300, Montgomery, TX, USA). Following incubation, cells were washed with ice-cold PBS and analyzed in FACS as described in the previous flow cytometry experiment. Binding of wtACE2 and ∆cytACE2 with spike S1-Fc proteins were quantified after subtracting the background of fluorescence intensity from pcDNA transfected cells. Binding of spike S1-Fc proteins with wtACE2 was normalized to 100%.

### 2.10. Production and Validation of Pseudotyped Coronaviruses (CoV PVs)

Pseudotyped SARS-1, SARS-2, and MERS-CoVs were produced as described elsewhere [21]. Briefly, full-length pCAGGS-MERS-S, pCAGGS-SARS-1-S, and pCAGGS-SARS-2-S were transiently expressed on HEK293T cells followed by VSVΔG/GFP infection. One-hour post-infection, cells were washed thrice with PBS and replaced with fresh infection medium DMEM-1% FBS (1% DMEM) containing VSV-G monoclonal antibody. Twenty-four hours post infection, cell supernatant containing pseudotyped coronaviruses (SARS-CoV-1-PV, SARS-CoV-2-PV, and MERS-CoV-PV) was harvested, pre-cleared of cell debris, and stored at −80 °C. The viral titer was determined by infection of Vero E6 cells after gradient dilution of the supernatant followed by counting the number of GFP-positive cells as described elsewhere [24,25]. Briefly, Vero E6 cells were seeded in a 96-well plate at 1 × 10^4^ cells per well and 100 µL of serially diluted (1:10) pseudoviruses were used for infection. One hour post incubation, cells were replaced with fresh 1% DMEM and were incubated for 24 h at 37 °C in a CO_2_ incubator. GFP-positive cells were counted manually thrice as well as after image acquisition using an Olympus CKX53 fluorescence imaging system followed by analysis using the cell-counting tool in ImageJ software.

To evaluate the specific receptor-mediated entry of pseudotyped coronaviruses, BHK21 cells were seeded in a 96-well plate and transfected with either pcDNA-ACE2, pcDNA-DPP4, or empty pcDNA plasmids. At 24 h post-transfection, pseudotyped viruses were infected on the transfected BHK21 cells and incubated for 1 h at 37 °C. One hour post infection, the cells were replaced with 1% DMEM. At 24 h post infection, GFP-positive cells were counted and relative infection was calculated with reference to mock.

### 2.11. Soluble ACE2 (sACE2) Blocking Assay

Soluble ACE2-blocking assay was performed as described elsewhere [26]. Briefly, serially diluted recombinant sACE2 protein (0.5 to 5 µg/mL) was preincubated with SARS-CoV-1-PV, SARS-CoV-2-PV, and MERS-CoV-PV at 37 °C for 1 h and the mixture was added independently to Vero E6 cells for 1 h. After infection, cells were replaced with infection medium and incubated for 24 h at 37 °C in a CO_2_ incubator. Twenty-four hours post-infection, GFP-positive cells were counted and plotted as percentage of infection.

### 2.12. Effect of Endocytosis Inhibitors on the Entry of CoV PVs

To test the effect of the inhibitors on pseudotyped coronavirus entry, a monolayer of Vero E6 cells was grown in a 96-well plate and the cells were treated independently with 20 mM ammonium chloride (Sigma Aldrich Cat No. A4514, St. Louis, MO, USA) or 100 µM Dynasore (Sigma Aldrich Cat No. D7693, St. Louis, MO, USA) diluted in 1% DMEM for 1 h at 37 °C [27]. Then, in the first set of experiments, the drugs were removed from the cells and infected with pseudotyped CoVs of MERS-CoV, SARS-CoV-1, SARS-CoV-2, and VSVΔG/GFP for 1 h. In the second set of experiments, in addition to pretreatment, cells were infected with pseudotyped CoVs along with the same concentration of the drugs. After 1 h infection, cells were replaced with fresh 1% DMEM and incubated for 24 h at 37 °C in a CO_2_ incubator. GFP-positive cells were counted and percentage of infection was calculated relative to mock-treated cells.

### 2.13. Colocalization of SARS-CoV-1 and -2 Spike S1-Fc with ACE2 Mutant Receptor

To analyze the colocalization of spike S1-Fc proteins of SARS-CoV-1 and -2 on wtACE2 and ∆cytACE2, BHK21 cells were grown on coverslips and transfected independently with 2 μg of wtACE2 and ∆cytACE2 plasmids. After 24 h incubation, cells were washed with 1× ice-cold PBS and incubated with 5 μg/mL spike S1-Fc protein for 1 h at 4 °C. Cells were fixed with 4% paraformaldehyde at RT and washed with 1× PBS. ACE2 expression was detected by immunostaining using goat anti-human ACE2 antibody for 1 h at 37 °C followed by incubation with Alexa Fluor 594-conjugated anti-goat IgG secondary antibody. Next, the spike S1-Fc proteins were detected by using goat anti-human IgG antibody conjugated with FITC (goat, 1:300, Bethyl, Cat No. A80119F, Montgomery, TX, USA). After 1 h incubation, cells were washed twice with PBS, mounted with VECTASHIELD, and visualized under the confocal microscope.

### 2.14. Receptor-Mediated Internalization Assay of CoV Spike S1-Fc and RBD-Fc Proteins

HEK293T cells were grown on coverslips at 1 × 10^5^ density per well and transfected independently with wtACE2, ∆cytACE2, and pcDNA plasmids. Following 24 h transfection, cells were incubated with 3 μg/mL of spike S1-Fc, RBD-Fc proteins of SARS-CoV-1, SARS-CoV-2, or KFDV envelope protein-Fc on wtACE2- or ∆cytACE2-transfected cells on ice for 30 min to allow the binding of proteins to the receptor, followed by internalization at 37 °C for 4 h. The results were compared to 0 h incubation at 37 °C, wherein the cells were processed for staining directly from 4 °C incubation. After incubation, cells were washed and fixed with 4% paraformaldehyde at RT. For the internalization staining, cells were washed twice with PBS and permeabilized with 70% ethanol and spike S1-Fc, or RBD-Fc proteins were detected by using FITC-conjugated goat anti-human IgG antibody for 1 h at 37 °C. After washing, S1-Fc and RBD-Fc internalization were analyzed using Zeiss LSM 880 confocal laser scanning microscope (Zeiss, Jena, Germany).

### 2.15. Colocalization of wtACE2 or ΔcytACE2 with SARS-CoV-1 and SARS-CoV-2 Spike S1-Fc Proteins

HEK293T cells were grown on coverslips and transfected with 2 µg of pcDNA3.1-ACE2, pcDNA3.1-∆cytACE2, or pcDNA3.1 independently. At 24 h post transfection, cells were treated with spike S1-Fc proteins (3 µg/mL) of SARS-CoV-1 or -2 on ice for 30 min followed by incubation at 37 °C for 4 h. After incubation, cells were washed twice with 1× PBS and fixed with 4% paraformaldehyde. Protein staining was performed in permeabilized or non-permeabilized cells to confirm the cytoplasmic localization of ACE2 and spike proteins. ACE2 expression was detected using goat anti-human ACE2 antibody (goat, 1:300, R&D, Cat No.AF933, Minneapolis, MN, USA) for 1 h at 37 °C followed by incubation with Alexa Fluor 594-conjugated anti-goat IgG secondary antibody (Rabbit, 1:500, Immunotag, Cat No. ITIF59418, St. Louis, MO, USA). Next, the spike S1-Fc proteins were detected using donkey anti-human IgG conjugated with DyLight 488 antibody (Donkey, 1:500, Invitrogen, Cat No. SA5-10126, Rockford, IL, USA). After 1 h incubation, cells were washed twice with PBS, mounted with VECTASHIELD, and visualized under the confocal microscope.

### 2.16. Infectivity of SARS-CoV-1 and -2 PVs in wtACE2- and ∆cytACE2-Expressing Cells

To investigate the role of the cytoplasmic domain of ACE2 in SARS-CoV-1 and -2 infection, BHK21 and HEK293T cells were transfected with either wtACE2 or ∆cytACE2 plasmids. After 24 h incubation, cells were infected for 1 h with SARS-CoV-1 and -2 PVs in the presence or absence of VSV-G or SARS-CoV antibodies. Fresh infection medium 1% DMEM was added to the cells and incubated for 24 h. The number of GFP-expressing cells was counted and the percentage of infection was calculated.

### 2.17. Statistical Analysis

All data are presented as mean ± SD using GraphPad Prism software version 9. Statistical significance was evaluated by one-way ANOVA analysis for comparisons between groups via GraphPad Prism software. * *p* < 0.05; ** *p* < 0.01; *** *p* < 0.001.

## 3. Results

### 3.1. ACE2 Cytoplasmic Domain Contains Conserved Endocytosis Motif

SARS-CoV-2 spike binds on the ACE2 receptor and enters the host cells. ACE2 has a short C-terminal cytoplasmic domain and a long extracellular domain. For membrane receptors, once the ligand binds on the extracellular domain, then the receptor conformation changes, followed by activation of cytoplasmic domain signaling for downstream pathways. Here, to know whether the cytoplasmic domain of human ACE2 is required for the activation of ACE2 upon spike binding, we analyzed the amino acid sequence of the cytoplasmic domain of ACE2 for any conserved phosphorylation sites or motifs. We found that residues 761–805 of ACE2 were relatively conserved between different species (Figure 1). Further, eight putative phosphorylation sites were predicted, which included T663, S776, Y781, S783, S787, N797, T803, S804, and a known internalization motif YXXΦ (X: any aa; Φ: hydrophobic aa), which are highly conserved among (non-)susceptible species (Figure 1). The YXXΦ motif is one of the four known motifs, YXXΦ, D/E XXXL L/I, FXNPXY, and YXXXΦN, that play a role in clathrin-mediated endocytosis, suggesting that YXXΦ might have a role in the internalization of SARS-CoV-2.

### 3.2. Surface Expression of Cytoplasmic Deletion Mutant of ACE2 Is Similar to Wildtype ACE2

Next, to know whether cytACE2 plays a role in the internalization of SARS-CoV-1 and -2, we generated plasmids expressing either full-length or complete cytoplasmic domain (43 aa)-deleted ACE2 (ΔcytACE2) (Figure 2A). These plasmids were transiently expressed on the non-susceptible human embryonic kidney (HEK293T) and baby hamster kidney (BHK21) cells. After 24 h incubation, mRNA transcript and protein expression of ACE2 and ΔcytACE2 were analyzed by RT-PCR, sequencing (Figure S2), and Western blot. As expected, ΔcytACE2 generated an mRNA transcript of 2283 bp and 105 kDa protein bands (Figure 2B), and the transcript and protein band sizes were slightly lesser with respect to the wildtype (wt) ACE2 (2420 bp and 110 kDa) (Figure 2B).

Receptor expression and proper presentation on the cell surface are critical for the binding of virus on host cells. The surface expression of ΔcytACE2 was analyzed by immunofluorescence (IF) and fluorescence-activated cell sorting (FACS) using an anti-ACE2 antibody. Surface presentation of ACE2 was detected in both wtACE2- and ΔcytACE2-transfected 293T cells (Figure 2C top panel), but not in pcDNA-transfected cells. However, the expression level was slightly lesser in ΔcytACE2 compared to wtACE2 (Figure 2D top panel). Similar results were observed in BHK21 cells as well (Figure S3A,B). The data clearly show that ΔcytACE2 expresses on the cell surface like wtACE2.

### 3.3. SARS-CoV-2 Spike S1-Fc Protein Binds on wtACE2 and ΔcytACE2

**Figure 3:**
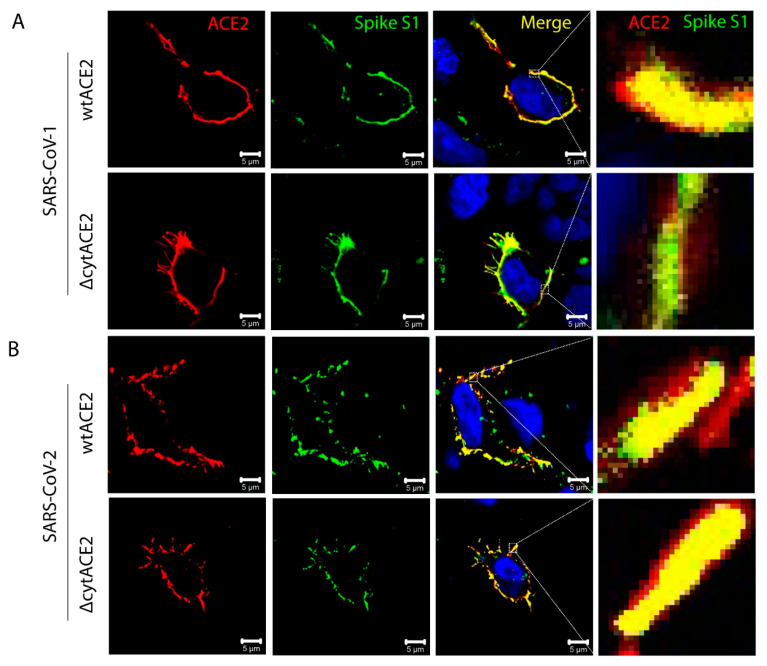
Colocalization of SARS-CoV spike S1-Fc proteins with wtACE2 and ∆cytACE2. (**A**) Wildtype and mutant plasmid constructs were transfected independently in BHK21 cells and 24 h post transfection, cells were incubated with spike S1-Fc proteins (5 µg/mL) of (A) SARS-CoV-1 and (**B**) SARS-CoV-2 at 4 °C for 1 h. Spike S1-Fc proteins were stained with FITC-labeled anti-human IgG-Fc (green) and ACE2 using goat anti-ACE2 antibody followed by Alexa Fluor 594-conjugated secondary antibody (red). Nuclei were stained with DAPI (blue). Colocalization of ACE2 and spike proteins is shown as merged panel in yellow.

To examine whether the removal of the cytoplasmic domain of ACE2 affects the binding of spike protein of SARS-CoV-2, we generated a recombinant dimeric S1 and RBD domain of spike glycoprotein C-terminally fused with the Fc region of human IgG (S1-Fc) (Figure S4A,B). Next, cells expressing wtACE2 or ΔcytACE2 were incubated with the recombinant S1-Fc proteins. The results show that SARS-CoVs S1-Fc bound on ΔcytACE2 like wtACE2, whereas pcDNA-transfected cells did not show any binding (Figure 2C, middle and lower panel). Moreover, no distinctions were seen in S1 binding when compared to wtACE2-S1 interaction (Figure 2D, middle and lower panel). However, the percentage of S1-bound positive cells correlates with the surface expression of ΔcytACE2 (Figure 2D, middle and lower panel) which clearly shows that the deletion of the cytoplasmic domain did not alter the spike S1 protein interaction with ACE2 extracellular domain. However, it is possible that S1 may bind non-specifically on the cell surface. To validate the direct interaction of ACE2 with SARS-CoV-1 and -2 S1-Fc, we performed an S1-ACE2 colocalization assay on wtACE2 or ΔcytACE2 expressing BHK21 cells. Both SARS-CoV-1 (Figure 3A) and SARS-CoV-2 (Figure 3B) spike proteins did bind on the head region of the ACE2 receptor and no dissimilarity was observed between the ΔcytACE2 and wtACE2. These data suggest that S1 precisely binds on ΔcytACE2 and wtACE2 receptors, not elsewhere on the cell surface.

### 3.4. Cytoplasmic Domain of ACE2 Is Not Necessary for Internalization of Spike S1 or RBD-Fc in HEK293T Cells

**Figure 4:**
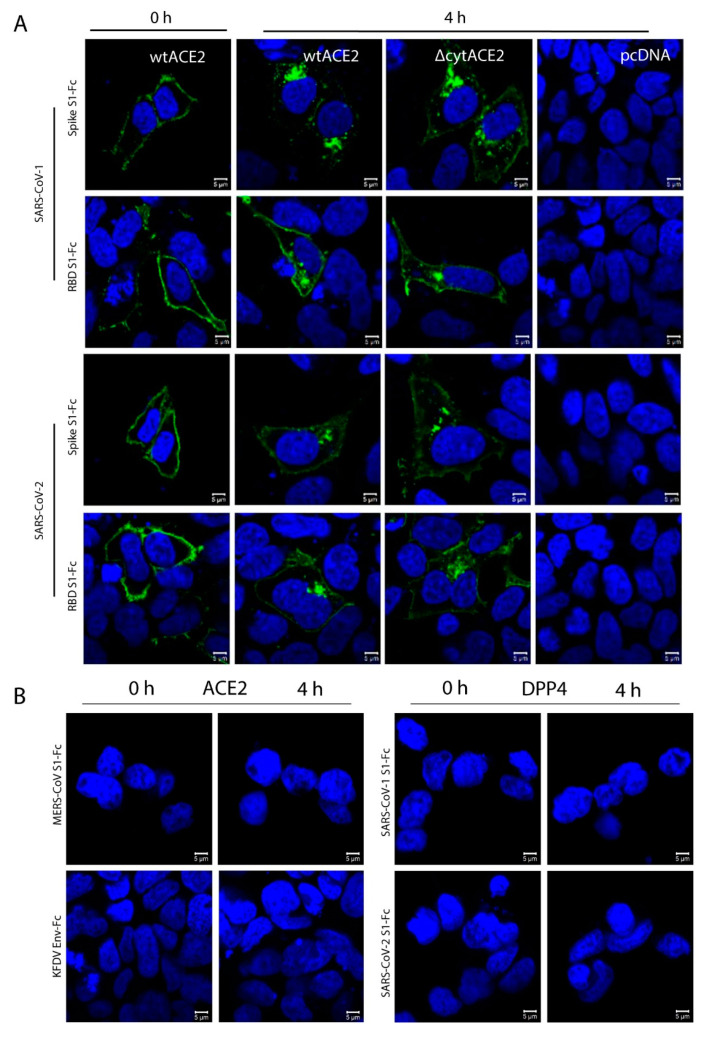
Cellular internalization of SARS-CoV-1 and -2 spike S1-Fc or RBD-Fc proteins. HEK293T cells were transfected independently with either wtACE2, ∆cytACE2, or pcDNA plasmids. Following 24 h transfection, cells were incubated for 0 or 4 h with (**A**) spike S1-Fc or RBD-Fc proteins (3 µg/mL) of SARS-CoV-1 or SARS-CoV-2 and then the S1-Fc proteins were stained with FITC-conjugated anti-human IgG and visualized under confocal microscopy. Similarly, (**B**) MERS-CoV S1-Fc or KFDV envelope protein Fc on wtACE2-expressing cells (left panel) and SARS-CoV-1 and -2 S1-Fc proteins on DPP4 (right panel). Nuclei were stained with DAPI (blue).

To check whether the interaction of spike proteins with ACE2 triggers any internalization signals via the cytoplasmic domain, HEK293T cells expressing wtACE2 or ΔcytACE2 were incubated with either 3 µg/mL of recombinant S1-Fc, RBD-Fc, or an Fc-fused envelope (E) protein of a flavivirus (Kyasanur Forest Disease virus—KFDV) (Figure 4). The protein was allowed to bind and internalize into the cells for 4 h at 37 °C, and the internalization was analyzed by confocal imaging. Not KFDV E-Fc, but both S1-Fc and RBD-Fc of SARS-CoV-1 and -2 were internalized in wtACE2 as well as ΔcytACE2-expressing cells. Nevertheless, no internalization was spotted in the empty plasmid-transfected cells. Further, to confirm the observed internalization was specific to ACE2, we expressed ACE2 on the surface of BHK21 cells and checked the internalization after treating with MERS-CoV S1-Fc (Figure 4B). Similarly, a binding assay was performed on DPP4 (MERS-CoV receptor)-expressing cells using SARS-CoV-1 and -2 S1-Fc proteins (Figure 4B). No attachment or internalization was seen in either of the conditions tested, which indicates that the internalization signals are specific to wtACE2 and ΔcytACE2. The translocated proteins were localized as puncta near the cell nucleus (Figure 4B) and the finding obtained by RBD internalization assay indicates that the RBD domain of SARS-CoV-1 and -2 alone is sufficient for triggering the internalization process.

**Figure 5:**
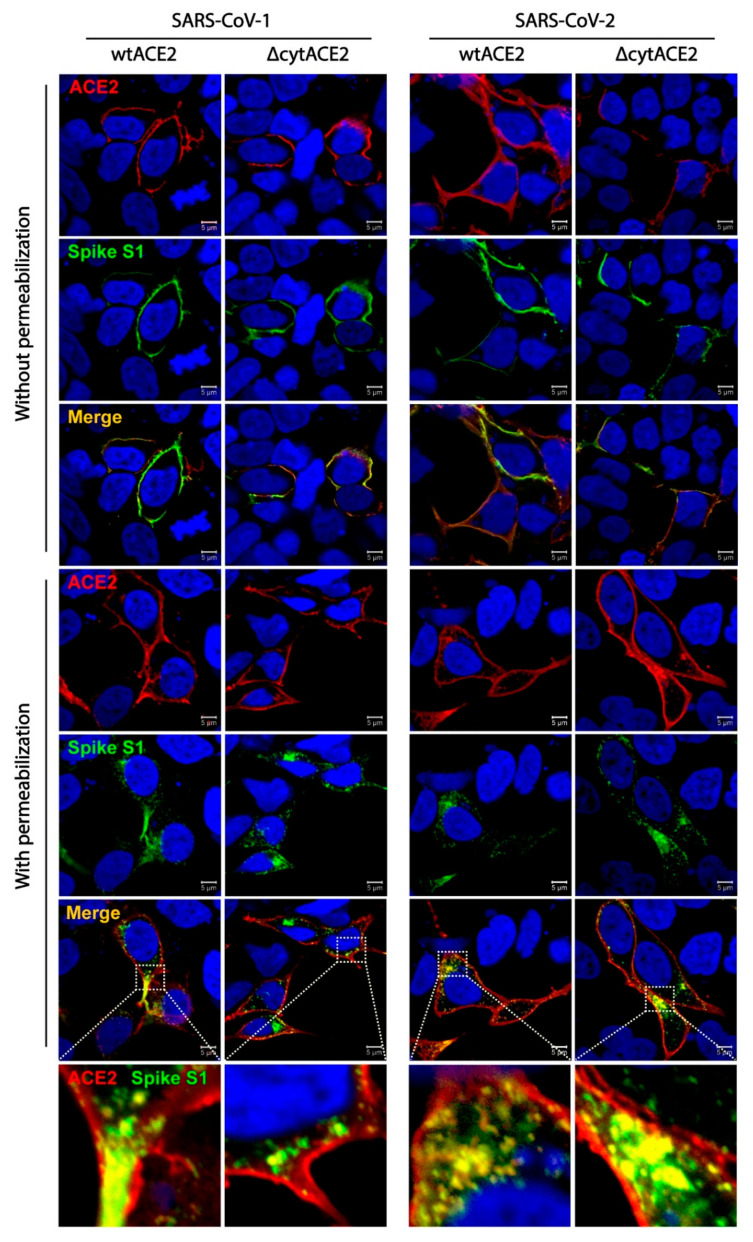
Cellular internalization of ACE2 and ∆cytACE2 along with SARS-CoV-1 or -2 spike S1-Fc proteins. HEK293T cells were transfected with wtACE2 and ∆cytACE2 independently and 24 h post transfection, cells were incubated with spike S1-Fc of SARS-CoV-1 and -2 for 4 h at 37 °C. To clearly distinguish between the internalized and surface-localized spike S1-Fc proteins, permeabilized and non-permeabilized cells were tested for internalization and colocalization. S1-Fc was stained using DyLight 488-conjugated anti-human IgG-Fc (green) and ACE2 using goat anti-human ACE2 antibody followed by Alexa Fluor 594-conjugated secondary antibody (red). Nuclei were stained with DAPI (blue). Colocalization of ACE2/∆cytACE2 and spike proteins during internalization shown as merged panel in yellow.

Next, to know whether the ACE2 receptor internalizes along with the spike proteins, we performed a colocalization assay followed by internalization of spike S1-Fc proteins of SARS-CoVs. Here, wtACE2- or ΔcytACE2-expressing HEK293T cells were subjected to ACE2 and S1-Fc staining with or without permeabilization. Surface colocalization of ACE2 and S1-Fc proteins were observed in non-permeabilized cells, whereas internalization of ACE2 along with S1 of SARS-CoV-1 and -2 were observed in permeabilized cells (Figure 5). These data show that SARS-CoV-1 and -2 spike proteins internalize to host cells along with their receptor.

### 3.5. SARS-CoV-1 and -2 Pseudotyped Coronaviruses Enter Cells via pH and Dynamin-Dependent Endocytosis

**Figure 6:**
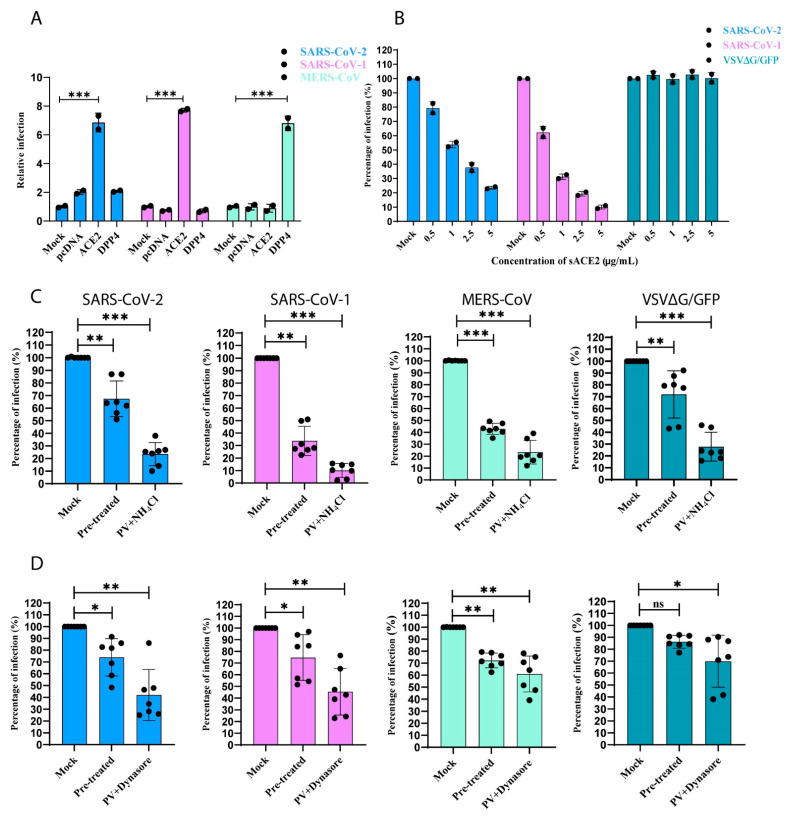
Receptor-mediated endocytosis of pseudotyped CoVs. (**A**) BHK21 cells expressing either ACE2, DPP4, or pcDNA were infected with SARS-CoV-1 and -2 PVs, MERS-CoV-PV, and VSV∆G/GFP. After 24 h, percentage of CoV-PVs infections were quantified by counting the number of GFP-positive cells. (**B**) SARS-CoV-1, -2, and VSV∆G/GFP were pre-incubated with varying concentrations of sACE2 (0.5 to 5 µg/mL) for 1 h prior to the infection on Vero E6 cells. After 24 h infection, cells expressing GFP were counted and the percentage of infection calculated. (**C**) Monolayer of Vero E6 cells were treated with either (**C**) 20 mM of NH_4_Cl or (**D**) 100 µM Dynasore 1 h prior (pre-treated) to the infection of PVs or either of the compounds were added to the cells during infection, followed by 1 h pre-treatment. The percentage of infection was calculated by counting the number of GFP-positive cells. Mock in each condition refers to untreated/non-transfected cells. The graphs represent data from at least three independent experiments. Data are shown as mean ± SD. *p*-value was determined by one-way ANOVA analysis (* *p* < 0.05; ** *p* < 0.01; *** *p* < 0.001).

**Figure 7:**
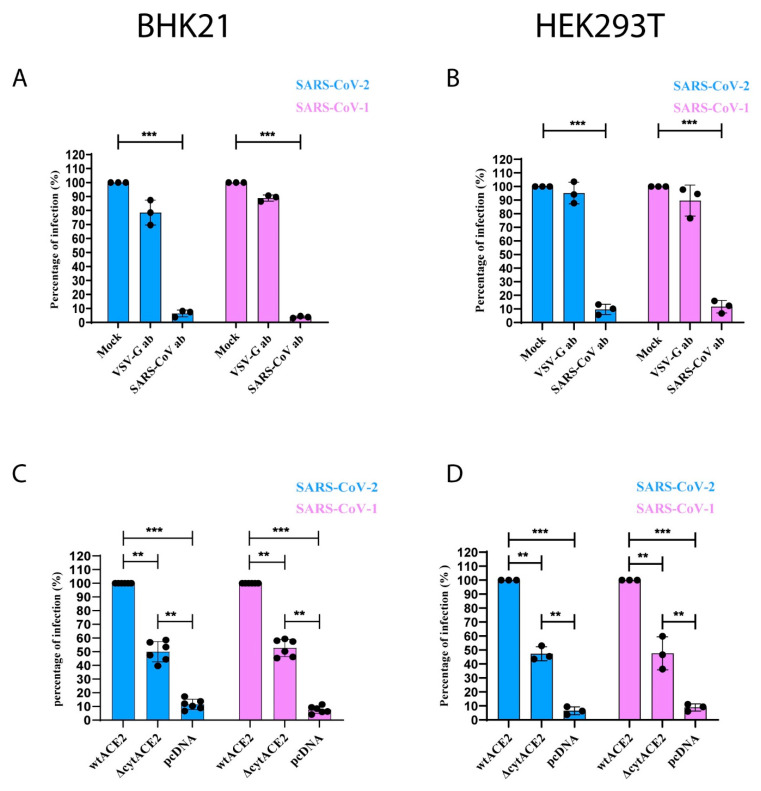
ACE2 cytoplasmic domain is not necessary for SARS-CoV-1 and -2 infection. (**A**,**B**) The presence of matured SARS-CoV-1 and -2 PVs were confirmed by neutralization assay using either SARS-CoV or VSV-G antibodies in wtACE2-expressing BHK21 or HEK293T cells. Pseudotyped SARS-CoVs were treated with SARS-CoV or VSV-G antibodies for 1 h followed by infection of BHK21 (left) or HEK293T (right) cells. (**C**,**D**) BHK21 and HEK293T cells were transfected with either wtACE2 or ∆cytACE2 receptors. Twenty-four hours post-transfection, cells were infected with pseudotyped SARS-CoVs for 1 h and infection of PVs was detected by counting GFP-positive cells. Mock in each condition refers to untreated/non-transfected cells. The graphs represent data from at least three independent experiments. Data are shown as mean ± SD. *p*-value was determined by one-way ANOVA analysis (** *p* < 0.01; *** *p* < 0.001).

To understand the role of ΔcytACE2 in the entry of SARS-CoV-1 and -2, first, we produced pseudotyped (PV) SARS-1, -2, and MERS-CoVs, and showed the receptor-mediated entry in BHK21 cells. Both SARS-CoV-1 and -2 PVs were permeable only in ACE2-expressing cells but not in DPP4 or empty plasmid-expressing cells (Figure 6A). Similarly, MERS-CoV PV enters only in DPP4 but not in wtACE2-expressing cells (Figure 6A). Further, the soluble form of ACE2 binds on the CoV-PVs and inhibits the entry in a dose-dependent manner, which clearly shows that SARS-CoV-1 and -2 PVs enter host cells through specific receptors (Figure 6B). Moreover, we evaluated the receptor-mediated endocytosis of the CoV-PVs using NH_4_Cl or Dynasore, known inhibitors of either pH or dynamin-dependent endocytosis. Significant inhibition of CoV-PVs infection was observed in cells pre-treated or treated during the viral infection with either of the compounds, suggesting that CoV-PVs enter the cells through receptor-mediated endocytosis (Figure 6C,D). In addition, anti-SARS-CoV-1 and -2 antibodies neutralized only SARS-CoV-1 and -2 PVs, but not VSV. In contrast, anti-VSV-G monoclonal antibody neutralized VSV but not CoV-PVs in wtACE2-expressing BHK21 and HEK293T cells, which indicates that the PVs are specific to the respective CoVs (Figure 7A,B).

### 3.6. Removal of Cytoplasmic Domain of ACE2 Does Not Affect SARS-CoV-1 and -2 Entry

**Figure 8:**
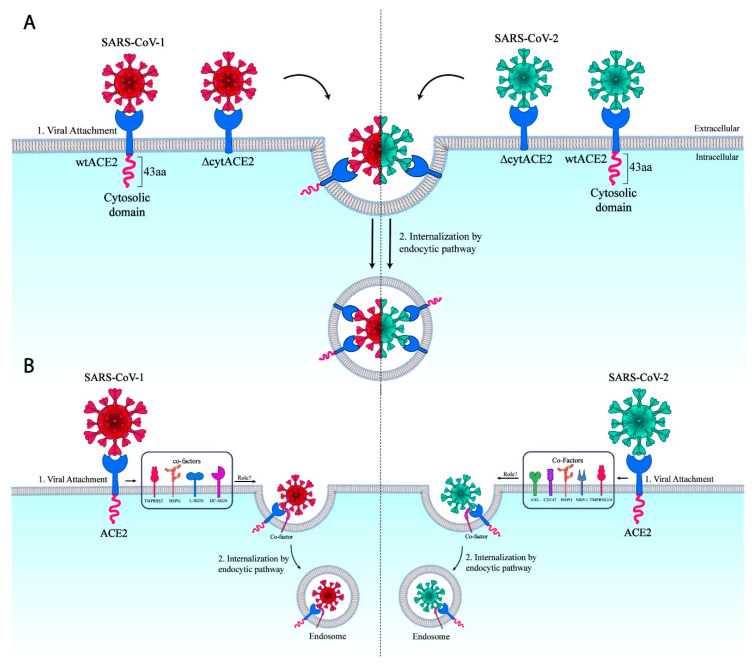
Schematic representation of ∆cytACE2-mediated entry of SARS-CoVs. (**A**) ACE2 cytoplasmic domain signaling independent internalization of SARS-CoV-1 and -2 into the cells. (**B**) Representation of co-factor/s which might be involved in the entry of SARS-CoV-1 and -2.

Next, we examined the role of the cytoplasmic domain of ACE2 in virus entry; BHK21 cells expressing either wtACE2 or ΔcytACE2 were infected with CoV-PVs, and the infection was calculated by counting the number of GFP-positive cells in the well. Interestingly, SARS-CoV-1 and -2 PVs infections were observed not only in wtACE2 but also in ΔcytACE2-expressing cells (Figure 7C). Similar experiments were performed on another non-susceptible HEK293T cell and found no distinction in infection between the cell types (Figure 7D), indicating that cytACE2 is not essential for the entry of SARS-CoV-1 and -2 (Figure 8A). The infection of CoV-PVs in ΔcytACE2-expressing cells correlates with the surface expression and S1 binding on ΔcytACE2 (Figure 2C,D). Altogether, the data suggest that the cytoplasmic domain of ACE2 is not essential for the entry of SARS-CoVs.

## 4. Discussion

Obligate intracellular pathogens, including coronaviruses, must enter into their host cells before beginning their life cycle. In general, entry of enveloped viruses into host cells occurs through two main pathways. Some viruses fuse their envelope directly into the plasma membrane and deliver the genetic material to the cytoplasm [28], whereas other viruses bind to the cell surface receptor/s and use clathrin- or caveolae-mediated host cell endocytic pathways to deliver the virion components to the cytoplasm [19]. In the endocytic pathways, virus envelope protein binds to the primary receptor, and subsequent attachment to the co-receptor/s followed by protease cleavages internalize the virus into the cytoplasm [29]. The recently emerged SARS-CoV-1 and -2 both use membrane-bound ACE2 as a primary receptor to enter the host cells [10,11].

SARS-CoVs spike attach to ACE2 and are then processed by membrane proteases, such as TMPRSS2/4, and enter into the host cell via endocytic pathways [14,30]. However, the virus internalization process post attachment/s to the receptor is not known for SARS-CoVs. Understanding the virus internalization process is essential to elucidate the SARS-CoVs’ transmission and pathogenesis, and for the eventual development of novel therapeutic interventions. It was believed that SARS-CoVs, post attachment to the receptor, enter the cell by internalization signaling cascade activated by the cytoplasmic domain of the receptor [20]. Recent studies, however, suggest that the endocytic pathways might be involved in the entry of SARS-CoV-1 and -2 [27,31]. There are four known internalization motifs, YXXØ, [D/E]XXXL[L/I], FXNPXY, and YXXXØN [32], that are involved in the receptor internalization and recycling processes. ACE2 has an internalization motif YXXØ in the cytoplasmic domain, which is conserved in (non-)susceptible species. We speculated that this might be essential for initiation and coordinating endocytosis of SARS-CoV-1 and -2. It was shown that YXXØ can bind to all four clathrin adapter protein (AP) complexes [33] and moreover, an AP2 inhibitor, sunitinib, reduces the infection of SARS-CoV-1, MERS-CoV, and SARS-CoV-2 [34]. Here, we found that the deletion of the complete cytoplasmic domain of ACE2 does not affect the entry of both SARS-CoV-1 and -2, suggesting that YXXØ motif or another phosphorylation site in the ACE2 cytoplasmic tail is not involved in the entry of SARS-CoVs in HEK293T and BHK21 cells.

It was shown that SARS-CoV-1 and -2 enter into the cells via the clathrin-mediated endocytosis pathway [27,31]. Host factors, including dynamin, which act as molecular scissors for newly formed vesicles originating from the plasma membrane, play a major role in the successful formation of endosomes and maturation of viruses [35]. Here, we found that treatment of a dynamin inhibitor (dynasore) or endosomal acidification inhibitor (NH_4_Cl) inhibited the entry of SARS-CoV-1 and -2, which is similar to other enveloped viruses including Chikungunya virus, Japanese encephalitis virus, reoviruses, and human immunodeficiency virus (HIV) [36,37,38,39]. These viruses utilize dynamin for cellular entry and exhibit reduced infection in the presence of dynasore [37,40,41,42].

A previous report revealed that SARS-CoV-1 infection does not require the cytoplasmic domain of ACE2 for internalization into the host cell [27]. Since SARS-CoV-2 binds to ACE2 and triggers the clathrin-mediated endocytosis, we investigated the role of the ACE2 cytoplasmic domain in the internalization of SARS-CoVs. We constructed an ACE2 mutant after removing the complete cytoplasmic domain and quantified the surface expression and binding efficiency with SARS-CoV-2 spike S1 proteins by FACS. ΔcytACE2 expresses on the surface of HEK293T and BHK21 cells and allows binding and colocalization of SARS-CoV-1 and -2 S1-Fc proteins. However, the binding of spike S1-Fc proteins correlates with reduced ΔcytACE2 expression compared to wtACE2. The results are consistent with previous studies, which shows that the surface expression of cytoplasmic tail- deleted membrane proteins SLC4-A11 and Platelet Endothelial Cell Adhesion Molecule (PECAM-1) are reduced compared to complete proteins [43,44]. Moreover, our internalization assay showed that recombinant spike S1 and RBD proteins were bound with wtACE2 or ΔcytACE2. ΔcytACE2-expressing cells exhibited successful internalization of SARS-CoV-1 and -2 spike S1 as well as RBD proteins like that of wtACE2, which is consistent with recent studies on SARS-CoV-1 RBD internalization into host cells [45]. Subsequently, we tested SARS-CoV-2 PV infection in wtACE2- or ΔcytACE2-expressing cells and the results were similar to SARS-CoV-1; the ACE2 cytoplasmic domain is dispensable for SARS-CoV-2 entry and infection. There are viral receptors which show the similar mechanisms upon virus binding. For instance, Dengue virus uses DC-SIGN to enter the cells. However, a recent study showed that, despite the fact that DC-SIGN contains two putative phosphorylation motifs in the cytoplasmic tail, Dengue virus (DENV) internalization is possible in the absence of DC-SIGN cytoplasmic domain [46].

In summary, the data clearly show that the ΔcytACE2 was properly synthesized and transported to the cell surface like wtACE2. We found that the ΔcytACE2 receptor was fully functional in the binding of CoV spike protein and mediated the entry of CoV-PVs. Thus, ACE2 receptor cytoplasmic domain signaling is not essential for the entry of SARS-CoV-1 and SARS-CoV-2. Recent studies show that tyrosine kinase inhibitors inhibit the entry of several enveloped viruses, including SARS-CoV-1, SARS-CoV-2, MERS-CoV, rabies virus, hepatitis C virus (HCV), Ebola, and Dengue viruses [34]. Moreover, several host elements have been identified as co-factors or alternative receptors for SARS-CoV-2 [14,15,16,17,47]. We speculate that the virus may bind to ACE2 and use a co-receptor/s or cellular elements that interact with the spike of SARS-CoV-2, including AXL, HSPG, CD147, NRP-1, or TMPRSS2/4 [14,15,16,17,47], to initiate the internalization (Figure 8B) like other enveloped viruses, such as porcine transmissible gastroenteritis virus (TGEV), influenza virus (IAV), porcine epidemic diarrhea virus (PEDV), herpes simplex virus 1 (HSV), and HCV [48,49,50,51]. Further studies on the usage of co-receptor/s or cellular factors that support the internalization of viruses can accelerate the invention of novel antiviral strategies.

## Data Availability

Not applicable.

## Supplementary Materials

The following are available online at https://www.mdpi.com/article/10.3390/cells10071814/s1, Figure S1: Representation of SARS-CoV-2 spike protein primary structure, Figure S2: Alignment of wtACE2 and ∆cytACE2, Figure S3: Cell surface localization and binding affinity with spike proteins of wtACE2 and ∆cytACE2 on BHK21 cells, Figure S4: Production and validation of spike S1-Fc and RBD-Fc proteins.
