# Supplementary material for: SARS-CoV-2 Cellular Entry Is Independent of the ACE2 Cytoplasmic Domain Signaling
Source: Cells. 2021 Jul 17;10(7):1814. doi: 10.3390/cells10071814 (PMC8304749; doi:10.3390/cells10071814)
Supplement: Supplementary file 1 [file cells-10-01814-s001.zip › cells-1279714-supplementary.pdf]

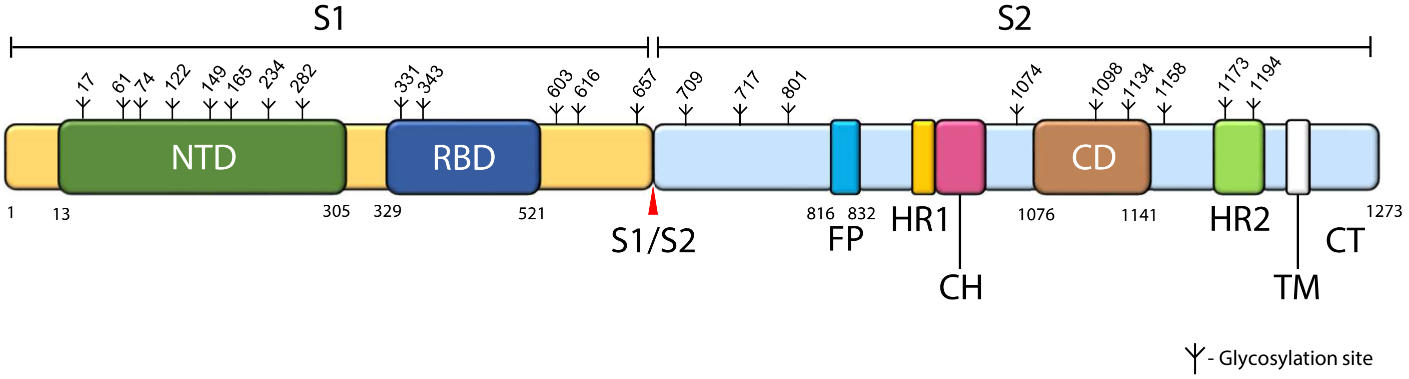

Figure S1. Representation of SARS-CoV-2 spike protein primary structure. Different domains are shown by different colours. NTD, N-terminal domain; RBD, receptor-binding domain; S1/S2, S1/S2 protease cleavage site; FP, fusion peptide; HR1, heptad repeat 1; CH, central helix; CD, connector domain; HR2, heptad repeat 2; TM, transmembrane domain; CT, cytoplasmic tail. The protease cleavage site is indicated by red arrow.

Supplementary Figure S2

```
                    2260      2270      2280      2290      2300
            ....|....|....|....|....|....|....|....|....|....|
hACE2       GGGAGTGATAGTGGTTGGCATTGTCATCCTGATCTTCACTGGGATCAGAG
ΔcytACE2    GGGAGTGATAGTGGTTGGCATTGTCATCCTGATC----------------

                    2310      2320      2330      2340      2350
            ....|....|....|....|....|....|....|....|....|....|
hACE2       ATCGGAAGAAGAAAAATAAAGCAAGAAGTGGAGAAAATCCTTATGCCTCC
ΔcytACE2    --------------------------------------------------

                    2360      2370      2380      2390      2400
            ....|....|....|....|....|....|....|....|....|....|
hACE2       ATCGATATTAGCAAAGGAGAAAATAATCCAGGATTCCAAAACACTGATGA
ΔcytACE2    --------------------------------------------------

                    2410
            ....|....|....|....
hACE2       TGTTCAGACCTCCTTTTAG
ΔcytACE2    ----------------TAG
```

Supplementary Figure S2. Alignment of wtACE2 and ΔcytACE2. WtACE2 and ΔcytACE2 were sanger sequenced and aligned using BioEdit software. The deletion in cytoplasmic domain of ACE2 is shown as gap in dashed line.

Supplementary Figure S3

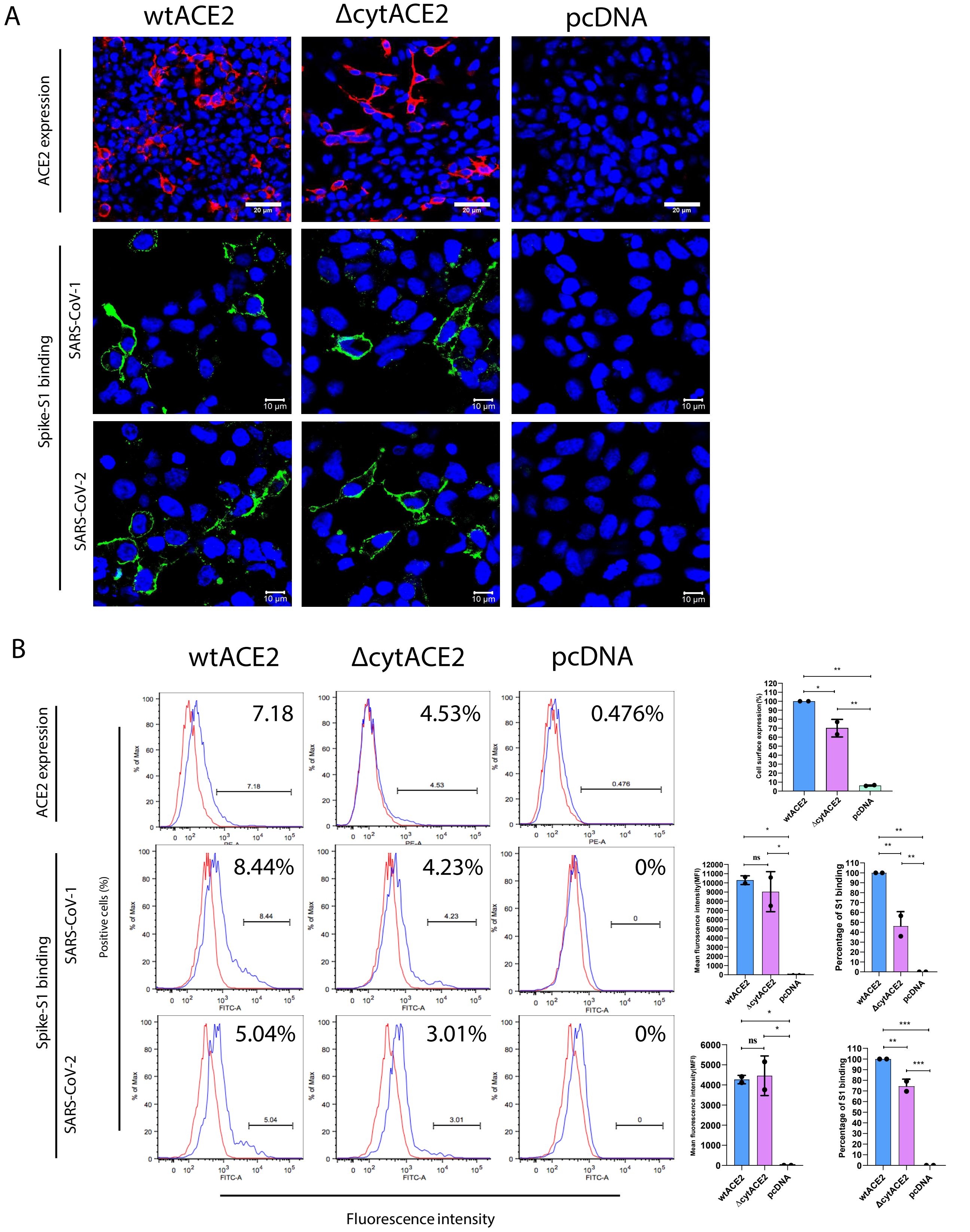

Supplementary Figure S3. Cell surface localization and binding affinity with spike proteins of wtACE2 and ΔcytACE2 on BHK21 cells. (A) wtACE2 and ΔcytACE2 plasmids were transfected in BHK-21 cells, after 24 h incubation cells were immunostained using anti-ACE2 antibody (red, upper panel). Cells expressing wtACE2 and ΔcytACE2 were treated with SARS-CoV-1 or -2 S1-Fc proteins (5µg/ml) for 1 h at 4 ºC. Surface binding of recombinant S1-Fc of SARS-CoV-1 (middle panel) and SARS-CoV-2 (lower panel) were detected with Goat anti-human IgG-Fc conjugated with FITC (green). Nuclei were stained with DAPI (blue). (B) Quantification of ACE2 expression (upper panel) and SARS-CoV-1 (middle panel) or SARS-CoV-2 (lower panel) S1-Fc bound wtACE2 or ΔcytACE2 expressing cells by FACS analysis. The graphs represent data from at least three independent experiments. Data are shown as mean ± SD. p value was determined by one-way ANOVA analysis (*$p < 0.05$; **$p < 0.01$; ***$p < 0.001$).

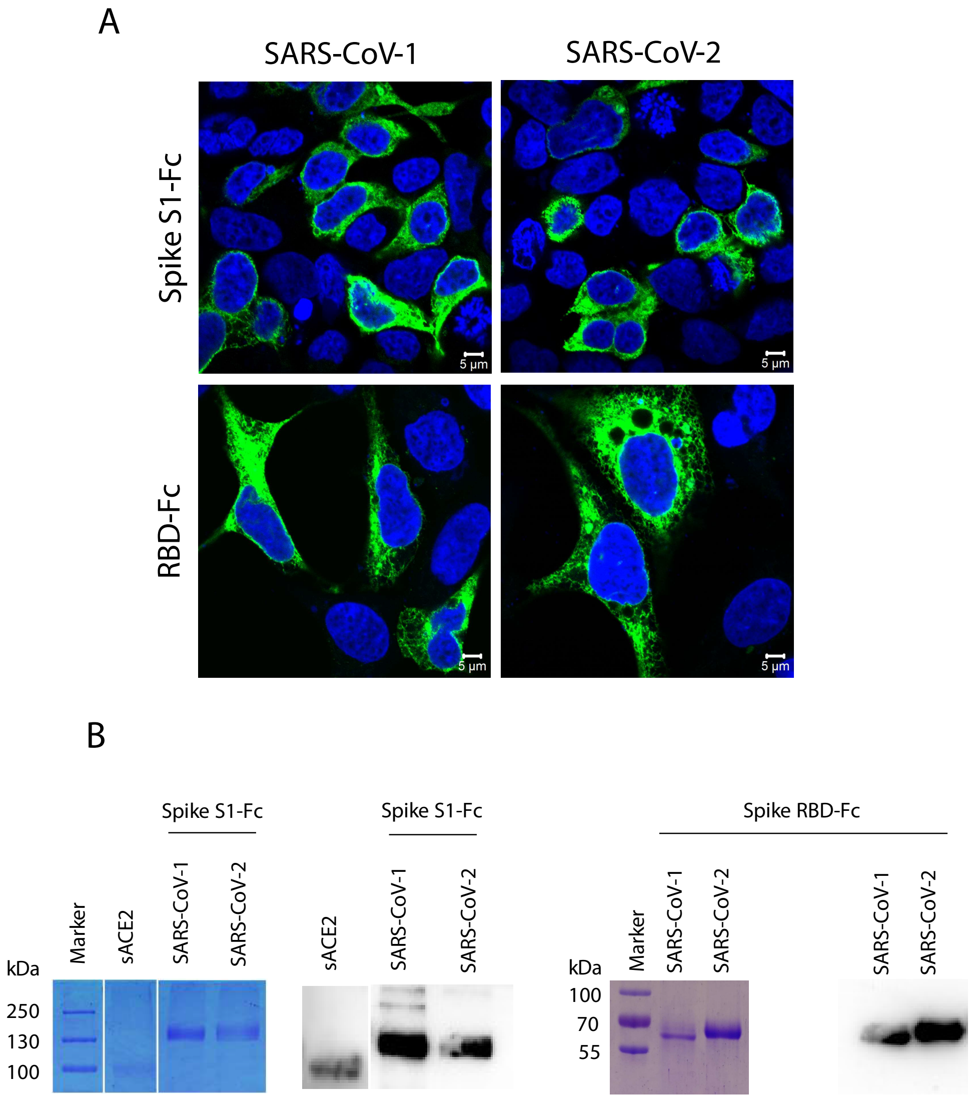

Figure S4. Production and validation of spike S1-Fc and RBD-Fc proteins. (A) HEK293T cells were transiently transfected with SARS-CoV-1 and -2 spike S1-Fc or RBD-Fc encoding plasmids. At 24 h post transfection, cells were fixed and permeabilized for staining with Goat anti-human IgG-Fc antibody conjugated with FITC (green). Nuclei were stained with DAPI (blue). Analysis of purified spike S1-Fc, RBD-Fc and soluble ACE2 (sACE2) proteins by (B) SDS-PAGE and western blot. Anti-human IgG conjugated with HRP was used to stain the Fc fused proteins and ACE2 antibody was used to stain sACE2 proteins. Protein molecular weight marker (kDa) is indicated on the left.
